# The Rise and Promise of Macrocyclic Hosts in Biomedicine

**DOI:** 10.1021/acscentsci.5c00891

**Published:** 2025-10-01

**Authors:** Yu-Chen Pan, Jia-Xuan Li, Dong-Sheng Guo

**Affiliations:** † College of Chemistry, State Key Laboratory of Elemento-Organic Chemistry, Key Laboratory of Functional Polymer Materials (Ministry of Education), Frontiers Science Center for New Organic Matter, Collaborative Innovation Center of Chemical Science and Engineering (Tianjin), 12538 Nankai University, Tianjin 300071, China; ‡ Xinjiang Key Laboratory of Novel Functional Materials Chemistry, College of Chemistry and Environmental Sciences, Kashi University, Kashi 844000, China

## Abstract

Macrocyclic hosts, possessing cyclic structures capable of encapsulating other molecules and ions, have exhibited unique characteristics in biomedical applications. Having preorganized cavities, macrocyclic hosts can monitor, intervene in, and simulate biological processes by complexing with bioactive substances. Additionally, the framework of macrocyclic hosts can exhibit remarkable efficacy in some applications such as transmembrane transport and antimicrobial. In this Outlook, we expressed some of our personal insights on the distinctive aspects of macrocyclic hosts in biomedical applications, highlighting some examples in disease diagnosis and treatment to provide illustrations. We also discussed the future opportunities and challenges of macrocyclic hosts in biomedical applications along with some suggestions on how to overcome these challenges. Considering the progress we have witnessed in the past decade, we believe that the future of this field is very bright.

## Graphical abstract

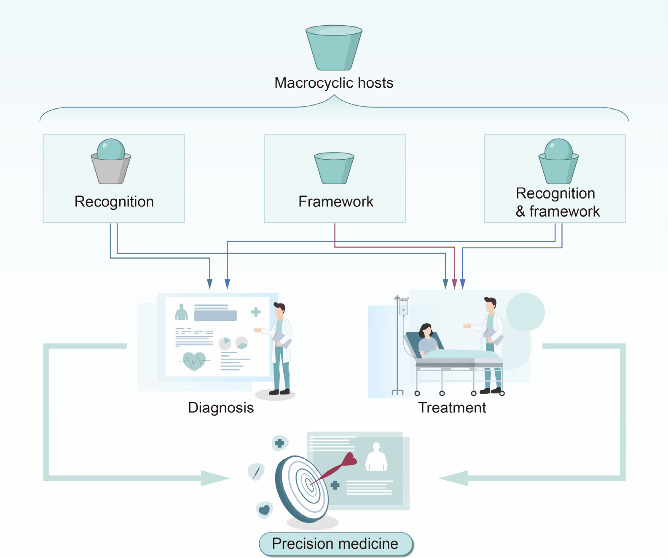

## 1 Introduction

Macrocyclic hosts are molecular entities containing a cyclic structure, capable of encapsulating other molecules or ions.^1^ In 1967, Pedersen described a vast array of cyclic polyethers, known as crown ethers (CEs), in his seminal publications.^2,3^ This marked the beginning of a flourishing development in the field of host–guest chemistry over the past nearly 60 years.^4^ Shortly thereafter, Profs. Cram and Lehn reported cavitands, spherands, and cryptands, sophisticated artificial hosts for metal cations and organic molecules.^5,6^ On account of their pioneering contributions to CEs, host–guest, and supramolecular chemistry, Pedersen, Cram, and Lehn shared the Nobel Prize in Chemistry in 1987. Actually, prior to the advent of wholly synthetic macrocyclic hosts (CEs), naturally occurring macrocycles had already been discovered and characterized. In 1891, Villiers described the isolation of a small amount of crystalline metabolic product, i.e. cyclodextrin (CD), following the incubation of *Bacillus amylobacter* in the presence of starch.^1,7^ Then in 1904, Prof. Schardinger characterized α-, β-, and γ-CDs, which possess hydrophobic cavities and capable of complexing hydrophobic guests.^1^ The discovery of CEs and CDs, along with their unique physicochemical properties and functions, has sparked strong interest in macrocyclic chemistry. Over the past several decades, a large number of artificial macrocyclic hosts, including calixarenes (CAs), cucurbiturils (CBs), and pillararenes (PAs), have been developed.^8–11^ Benefiting from the preorganized recognition sites and unique framework, they have made substantial advancements in various fields. For example, CEs can form organophilic complexes with metal cations, transferring them from the solid or aqueous phase to the organic phase, thereby being applied as phase-transfer catalysts.^12^ CDs can complex active substances and improve their stability and solubility, thereby have been widely used as food and pharmaceutical additives.^13,14^ Alkylated CA derivatives exhibit excellent surface activity, serving as demulsifiers in petroleum extraction to improve the speed of oil–water separation and increase crude oil recovery rates (https://oilfield.siigroup.com/calixarene.asp#).

The development of macrocyclic chemistry has been greatly inspired by biological processes, such as the protein–ligand recognition and the nucleic acid assembly, which have significantly promoted the diversity in the architectures and functions of macrocyclic host-based systems.^15^ As a reciprocation to life systems, biomedical application has become a significant functional output of macrocyclic chemistry. Macrocyclic hosts have also exhibited unique charm in this field, demonstrating some unparalleled advantages.^7,16,17^ First, macrocyclic hosts have preorganized cavities, which is the prominent characteristic compared to other biomedical materials. Molecular recognition is the basis of numerous biological processes and is also a powerful technology for diagnosis and treatment of diseases.^18,19^ For example, upon recognizing biomarkers, we can detect their types and concentrations, thereby serving in disease diagnosis.^20^ Upon recognizing pathogenic molecules, we can intervene in their effects on the body, thereby treating related diseases.^21^ Second, the frameworks of macrocyclic hosts can also exhibit biological activities, and some of them are inaccessible or incomparable to other small molecules.^22^ For example, due to the suitable size and 7-fold symmetrical structure, β-CD can effectively block the pore of the channel protein PA_63_ of *Bacillus anthracis*, thereby alleviating the toxicity of *Bacillus anthracis*.^23^ Third, macrocyclic hosts have precise molecular weights and well-defined structures, ensuring good batch-to-batch reproducibility, which is critical for quality control.^24^ Moreover, they have high chemical and thermal stabilities, and thus are expected to resist degradation byproducts, thereby avoiding the toxicity that may be caused by byproducts and ensuring effectiveness.^25^ Finally, macrocyclic hosts also exhibit broad modification tunabilities, rendering them adaptable to a wide range of application cases.^16,26^ Based on these advantages, more than 90 clinical trials based on macrocyclic hosts exist (www.clinicaltrials.gov), and more than 35 macrocyclic host-containing drugs are available on the market.^27^

Herein, we express some of our personal views on the biomedical applications of macrocyclic hosts. We have selected some examples, focusing mainly on disease diagnosis and treatment, to illustrate our points on the uniqueness of macrocyclic hosts in these applications as well as the future opportunities and challenges they face. However, the biomedical applications of macrocyclic hosts extend far beyond the diagnosis and treatment of diseases. They also include facilitating protein crystallization, catalytic synthesis and separation of small molecule drugs, and so on.^28–30^ The examples presented here are just the tip of the iceberg. For a more comprehensive and detailed understanding of the field, we recommend some recent excellent review articles.^31–37^

## 2 Characteristics of Macrocyclic Hosts

Before discussing the biomedical applications of macrocyclic hosts, we introduce them briefly so that readers who are unfamiliar with them can also understand their functions well. Macrocycles are defined as molecules and ions containing a ring of 12 or more atoms, while macrocyclic hosts are macrocycles that can accommodate other molecules within their rings. Currently, over 50 types of macrocyclic hosts have been reported, greatly propelling the development of supramolecular chemistry.^38^ Because of space limitations, here we only introduce a few macrocyclic hosts, those mentioned in the following examples.

CDs are composed of α-1,4 glycosidic bond linked oligosaccharides (Scheme 1). They have hydrophilic external surface and hydrophobic hollow cavity, due to the hydroxyl groups of the glucose units are oriented toward the outside at the orifice of the two ends while methinic protons are located inside the cavity.^7^ Therefore, CDs primarily bind alkyl/aryl residues and anionic molecules by providing hydrophobic interaction and hydrogen bonding.^39^ As one kind of natural compound, CDs exhibit excellent water solubility and biocompatibility, leading to their extensive applications in biomedicine.^40^

**Scheme 1:**
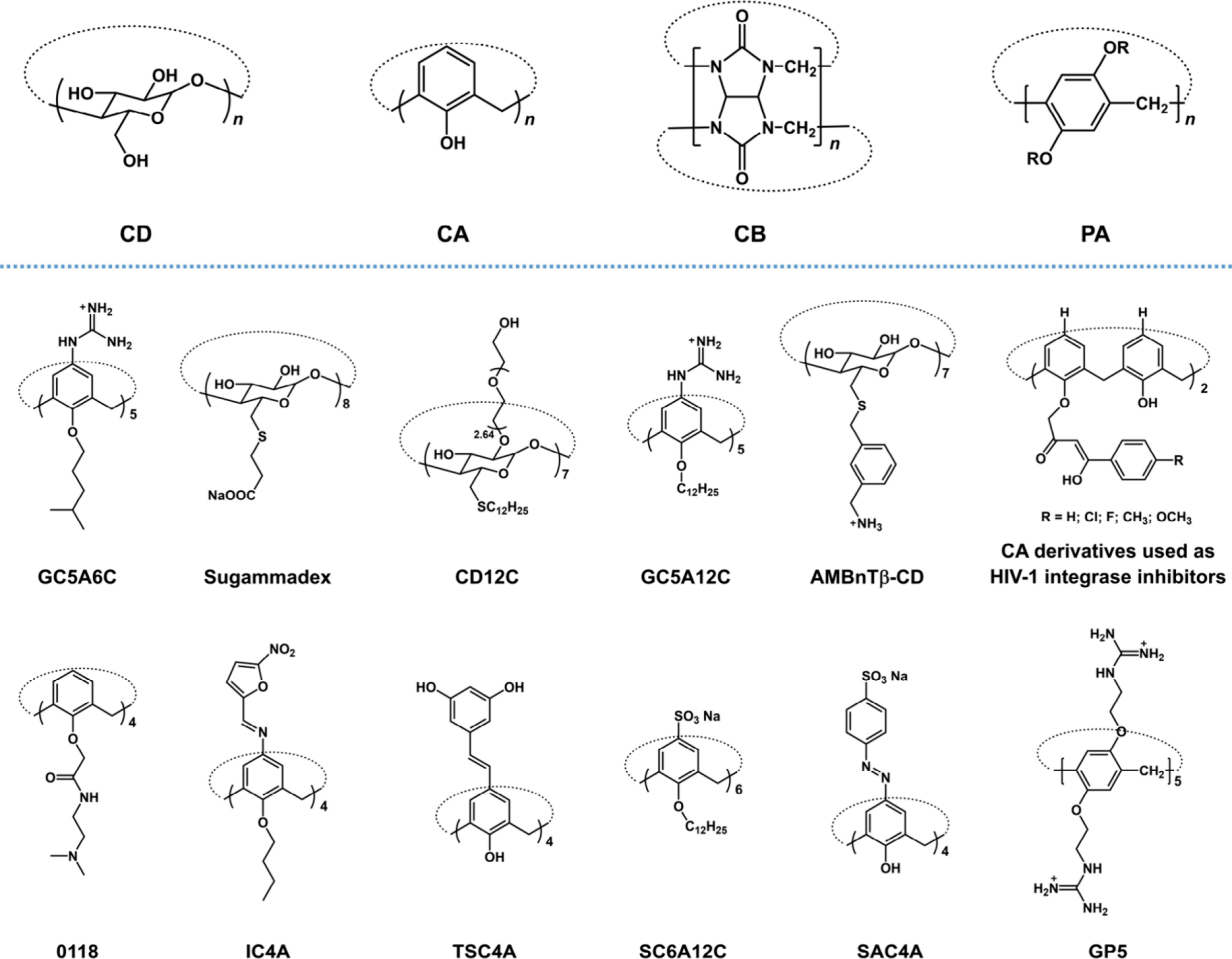
Chemical Structures of CD, CA, CB, PA, and Some Macrocyclic Host Derivatives Mentioned in This Outlook

CAs are macrocycles formed by phenolic units linked by methylene groups at positions 2 and 6, possessing hydrophobic and electron-rich cavities (Scheme 1).^41^ The cavities of unsubstituted CAs are shallow and exhibit poor recognition properties. Fortunately, CAs are ease of modification. All of their upper rim, lower rim, and methylene bridge can be derivatized, and asymmetric modifications can be easily achieved as well.^16^ Through appropriate modifications, we can adjust the recognition properties and toxicity of CAs, and impart various functionalities to them.^18^

CBs consist of glycoluril units connected by methylene groups, showing a pumpkin-like shape with two hydrophilic carbonylated rims and a hydrophobic cavity (Scheme 1).^42^ CBs can provide hydrophobic, ion-dipole, and hydrogen bonding interactions, making them suitable for complexing neutral and positively charged guests. What makes CB most attractive is its exceptionally strong binding to certain guests, even reaching 10^17^ M^–1^ in binding affinity.^43^ However, the difficulty in derivatization and lack of sufficient water solubility limit the biomedical application of CB to some extent.

PAs have a similar structure with CAs. They are formed by phenyl units linked by methylene groups at positions 2 and 5, and also possess hydrophobic and electron-rich cavities like CAs (Scheme 1).^44^ Different from CAs, the cavities of PAs are in pillar shape, making them highly suitable for applications involving ions/water channels.^45^ Additionally, PA is chiral and typically exists as racemates, making the isolation of enantiomerically pure PA very challenging, which can be a double-edged sword for biomedical applications.

## 3 Biomedical Applications of Macrocyclic Hosts

As previously mentioned, the preorganized recognition sites and unique framework of macrocyclic hosts endow them with various functions in the field of biomedicine. Here, we provide a brief overview of their roles in disease diagnosis and treatment, focusing on highlighting their unique characteristics. Our introduction will be structured around the ways in which the macrocyclic hosts exert their functions: through recognition, through the framework, and through both recognition and framework (Scheme 2).

**Scheme 2:**
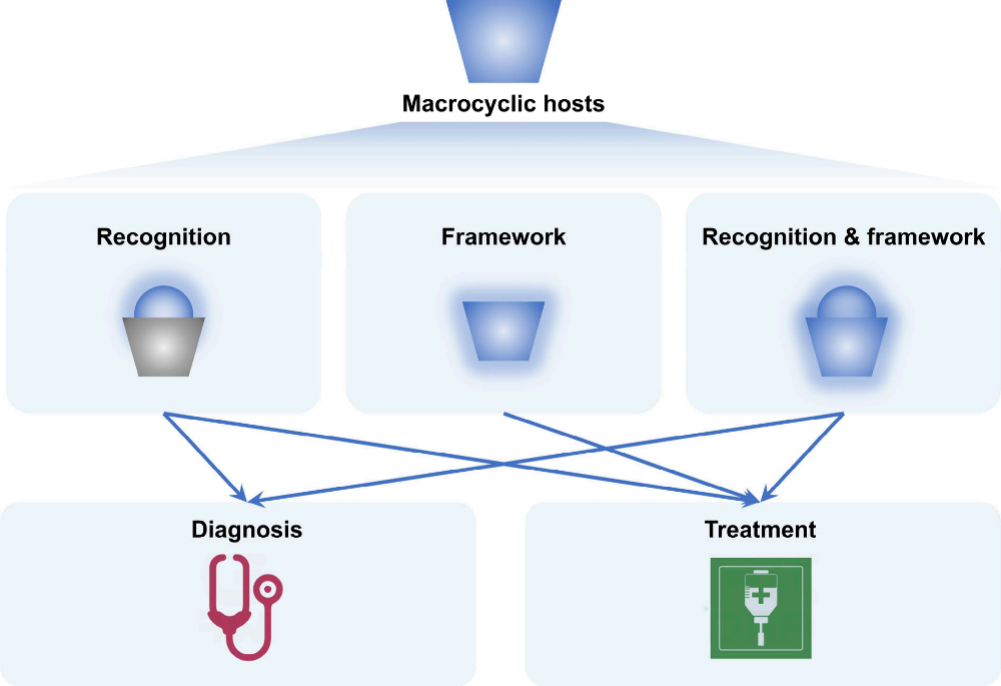
Ways through Which Macrocyclic Hosts Exert Their Biomedical Functions

### 3.1 Exerting Functions through Recognition

#### 3.1.1 Diagnosis

Sensing biomarkers in bodily fluids, such as blood and urine, is a crucial approach for disease diagnosis, with the key lying in the high-sensitivity capture of biomarkers. In single sensing, the receptor is designed and optimized for a particular biomarker based on the “lock and key” principle, and is expected to have strong and selective affinity to the biomarker.^46^ Strong interactions lead to low detection limits for biomarkers, ensuring the sensitivity of diagnosis. Selective interaction prevents interference from other coexisting substances, ensuring the accuracy of the diagnosis. Macrocyclic hosts have preorganized recognition sites and adjustable cavity size and ease of introducing additional anchoring points, offering the potential to achieve satisfactory recognition. Additionally, macrocyclic hosts can significantly impact the fluorescence of commercial dyes, facilitating the conversion of recognition events into easily detectable signals.^47^ For example, to sensing lysophosphatidic acid (LPA), a biomarker of ovarian cancer, the guanidinium-modified calix[5]­arene (GC5A6C, Scheme 1) was designed.^48^ The calix[5]­arene was screened because of its optimal cavity size, allowing the alkyl chain of LPA to thread through while not being too large, thus ensuring sufficient rigidity. Guanidinium groups were decorated at the upper rim of CA to donate charge-assisted hydrogen bonds (salt bridge) with the phosphate head of LPA. Alkyl chains were attached at the lower rim to provide a hydrophobic interaction with the tail of LPA besides rigidifying the CA conformation. Collectively, GC5A6C showed high binding affinity with LPA with the binding constant of 1.6 × 10^8^ M^–1^ (Figure 1a). Common interferents like amino acids and carbohydrates do not have strong affinity with GC5A6C, thus have minimal impact on the sensing system. Thanks to the strong and selective binding, the detection limit in untreated serum can be as low as 1.7 μM, meeting the requirements for early diagnosis of ovarian cancer (Figure 1a). Macrocyclic hosts can also strongly and selectively bind to many other biomarkers, such as tryptophan, norepinephrine, and trimethylamine N-oxide, promoting early and precise diagnosis of corresponding diseases.^49–51^ Nevertheless, macrocyclic hosts are not proficient in specific recognition. Achieving high selectivity in binding to biomarkers can be quite challenging for them. It requires a two-way effort. On one hand, meticulous design of the macrocyclic host is essential. On the other hand, biomarkers themselves should possess easily recognizable sites, appropriate size and shape, and be devoid of similar interfering substances. To address this issue, Prof. Nau proposed the Supramolecular Tandem Assay, leveraging the specificity of enzymatic reactions to complement the lack of specificity in macrocyclic recognition.^52^ This strategy has shown success in sensing enzymes and enzyme substrates.^53,54^

**Figure 1:**
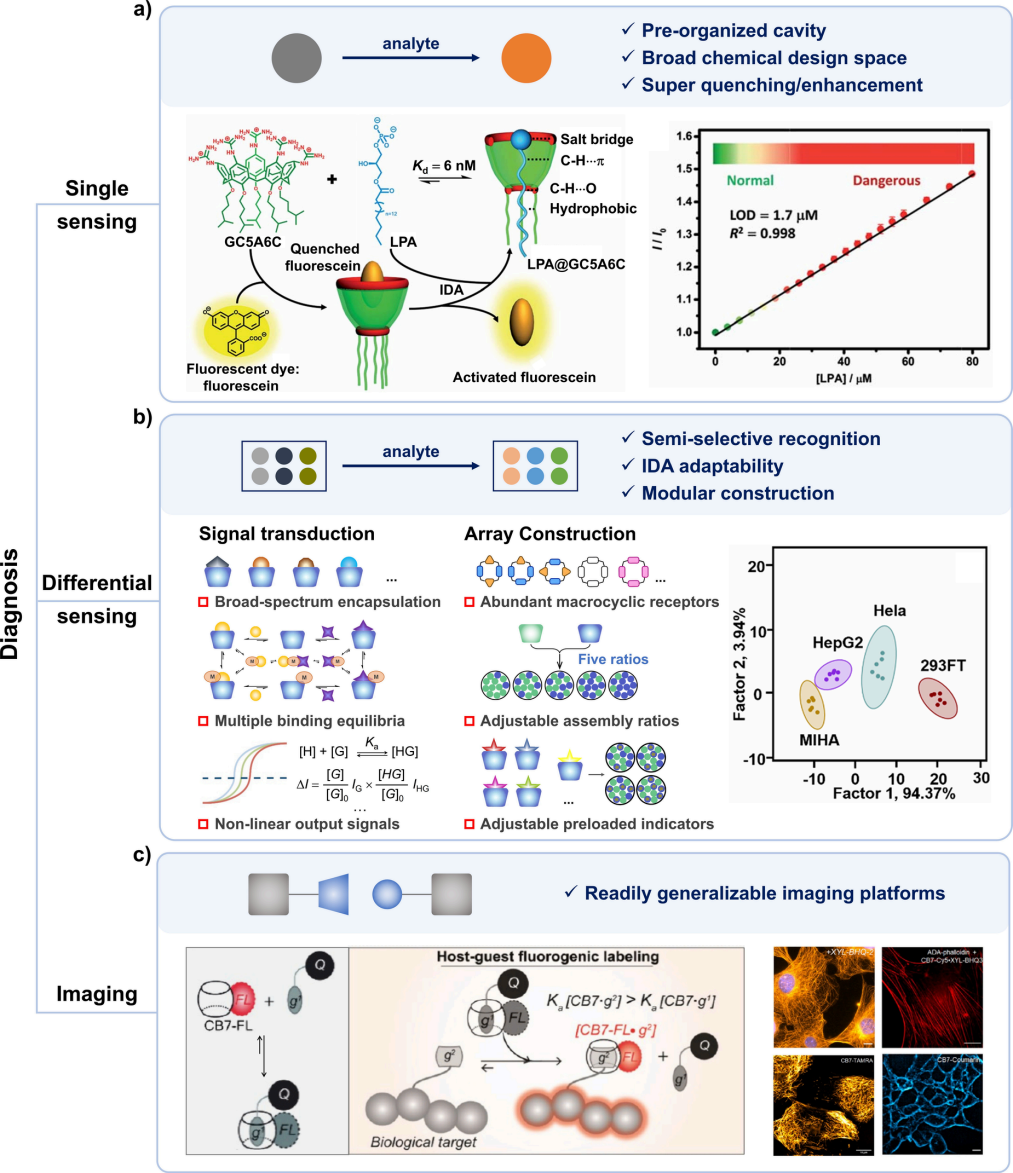
Applications of macrocyclic hosts in disease diagnosis through single sensing, differential sensing, or imaging. (a) Schematic diagram of single sensing (upper) and an example of using GC5A6C to achieve the fluorescence “switch-on” sensing of LPA (bottom). Adapted with permission from ref 48. Copyright 2018 The authors, some rights reserved; exclusive licensee Royal Society of Chemistry. Distributed under a Creative Commons Attribution 3.0 Unported License. (b) Schematic diagram of differential sensing (upper) and the advantages of macrocyclic hosts in the signal transduction and array construction (left bottom). The successful discrimination of different cell lines using macrocyclic host-based sensing array was shown on the right bottom. Adapted with permission from refs 59 and 63 . Copyright 2017 Wiley-VCH GmbH. Distributed under the terms of Creative Commons Attribution license. Copyright 2022 The authors, some rights reserved; exclusive licensee Royal Society of Chemistry. (c) Schematic diagram of the construction of readily generalizable imaging platform based on macrocyclic hosts (upper), and an example of using CB derivatives to construct biorthogonal fluorogenic imaging platform (bottom). Adapted with permission from ref 68, Copyright 2024 American Chemical Society.

Compared to highly selective recognition tools like antibodies (which almost exclusively bind to target molecules), the recognition by macrocyclic hosts is “semi-selective”. This means that they can complex a wide range of guests, while their binding affinities is influenced by structural differences among the guests (https://suprabank.org). This character makes macrocyclic hosts particularly suitable for applications in differential sensing. Differential sensing, inspired by the olfactory systems in mammals, utilizes the cross-reactivity of multiple sensor units toward multiple components (sometimes a single component) within the system to be analyzed to generate a distinctive fingerprint for the system.^46^ The sensing efficacy heavily relies on the quality and quantity of sensor units. Macrocyclic hosts are powerful tools for constructing high-quality sensor units (Figure 1b). First, the semiselective recognition ability endows them to bind to multiple compounds, thereby promoting cross-reactivity, which is crucial for increasing discrimination index. For example, Profs. Biedermann and Kappes reported a sensor array based on the CB derivative that distinguished over 10 bioorganic analytes.^55^ This success was attributed to the binding capability of the CB derivative with all these analytes, each exhibiting varying affinities. Second, macrocyclic hosts can modulate the fluorescence of commercial dyes through complexation, thereby their recognition behavior can be converted into detectable signals by the indicator displacement assay (IDA).^56^ The presence of multiple equilibria during the IDA process can also enhance the cross-reactivity. For example, Profs. Zhong and Hooley constructed a sensor array by deep cavitands and fluorescent dyes for differentiating G-quadruplex topologies.^57^ They emphasized the importance of multiple equilibria, including the complexation equilibriums of both binary complexes (DNA-dye and receptor-dye) and ternary complexes ((DNA-dye)-host and DNA-(dye-host)) for enhancing the discrimination results. Additionally, macrocyclic hosts can construct sensor arrays in a modular fashion, which also offers advantages in increasing the quantity of sensor units (Figure 1b).^58^ The broad-spectrum recognition capability of macrocyclic hosts allows for the easy construction of numerous sensor units simply by changing the types and concentrations of commercial dyes. Moreover, amphiphilic macrocyclic hosts can coassemble to form heteromultivalent recognition materials, where the types and ratios of assembled hosts determine the varied recognition performance of the coassembly. Therefore, a large number of sensor units can be obtained by varying the types and ratios of the amphiphilic macrocyclic hosts. For example, we assume that one dye can only be complexed with one macrocyclic host, then by using two amphiphilic hosts, five different coassembly ratios, and five host/dye ratios, a 50-sensor unit library can be built (2 indicators × 5 coassembly ratios × 5 host/dye ratios).^58^ Benefiting from above advantages in both signal transduction (enhancing cross-reactivity) and array construction (increasing sensor unit quantity),^59^ macrocyclic hosts have been widely used for differential sensing, enabling the discrimination of cancer cells, bacteria, proteins, peptides, etc., thereby contributing to the diagnosis of diseases such as cancer and bacterial infections (Figure 1b).^60–63^

Imaging of lesion sites is an in situ diagnostic method that can provide critical information such as the location and size of the lesions, offering valuable references for treatment. However, compared to in vitro sensing, in vivo imaging poses more challenges. A bioimaging system should exhibit good biocompatibility, possess lesion targeting capability, effectively handle interference from complex coexisting substances, and incorporate noise reduction features such as stimulus-responsive activation.^7,16^ Macrocyclic hosts have also achieved significant advancements in bioimaging, including imaging of tumor sites, injured kidneys, etc.^64–67^ In numerous applications, we believe that one major appeal lies in the advantage of macrocyclic hosts in constructing readily generalizable imaging platforms. This serves as a perfect manifestation of the modular characteristics of supramolecular chemistry. Prof. Agasti reported a bio-orthogonal fluorogenic imaging strategy based on CBs, which can be applied for imaging with dyes of different emission wavelengths and targeting different tissues (Figure 1c).^68^ Bio-orthogonal fluorogenic imaging, in which the fluorescence dye unmasks fluorescence signals in response to bio-orthogonal reactions, presents a promising imaging technique as it eliminates the need to wash out excess dyes. A convenient strategy to construct a bio-orthogonal imaging system is to connect a fluorescence dye with a quencher, subsequently inducing the dissociation of the dye-quencher pair at the location of interest to restore fluorescence. However, selecting a suitable connection method and dissociation trigger that is applicable to multiple dyes and various tissues and remains undisturbed by other substances within the biological system is highly challenging. To address this challenge, Prof. Agasti proposed a supramolecular guest exchange strategy.^68^ The fluorescence dyes were linked to CB7 covalently, and quenchers were linked to the guest (g^1^). The complexation of CB7 and g^1^ led to the quench of dyes. Then a higher-affinity guest g^2^ was covalently linked to the specific biological target of interest in location. When the quencher-g^1^@dye-CB7 pair reached the location of interest and encountered g^2^, g^2^ displaced quencher-g^1^ from the cavity of dye-CB7, leading to the recovery of fluorescence (Figure 1c). The authors validated the universality of this strategy by using 7 dyes with emission wavelengths ranging from 450 to 675 nm and imaging various sites including thoracic muscle tissue, cell membrane, and microtubules. In addition, Prof. Wang has developed a readily generalizable drug/dye delivery strategy by integrating the macrocyclic hosts with targeted living cells/nanoparticles and integrating guests with drugs/dyes.^69,70^ The recognition between the host and guest facilitated the “marriage” between drugs/dyes and targeted cells/nanoparticles, leading to the accumulation of drugs/dyes at the lesion location. For example, through the recognition of CB7 and ferrocene, ferrocene-capped gold nanoparticles can be precisely delivered to CB7-capped iron oxide nanoparticles, that were first magnetically deposited into the tumor as an artificial target, thereby promoting the computed tomography imaging of the tumor.^71^

#### 3.1.2 Treatment

The macrocyclic hosts provide a pharmacokinetic (PK) approach to treating diseases. In this approach, a host is employed to sequester pathogenic molecules, preventing them from causing harm. Compared with the pharmacodynamic approach, which focuses on designing an antagonist that binds the corresponding receptor in the body tightly to block the interaction between the pathogenic substance and the receptor, the PK approach is more straightforward and simpler. It does not require precise knowledge of the biological mechanism of action or the 3D structure of the biomolecular receptor.^72^ The therapeutic efficacy can be predicted by measuring the binding affinity between the therapeutic agent and the pathogenic substance, thereby saving both time and money. Among numerous macrocyclic sequesters, one of the most well-known is the Sugammadex (Scheme 1), a γ-CD derivative marketed by Merck as Bridion. Sugammadex is a therapeutic agent for the reversal of neuromuscular blockers rocuronium, by forming a tight host–guest complex with rocuronium (1.8 × 10^7^ M^–1^ in water).^73^ The reverse effect was verified in vivo in guinea pigs, cats, Rhesus monkeys, and finally humans (Figure 2a). Bridion was first approved by the European Union in 2008 and then by the United States FDA in 2015. Global sales of Bridion in 2024 amounted to $1.76 billion. Besides neuromuscular blockers, macrocyclic hosts have also been used to capture and sequester the toxicity of other exogenous drugs/toxins and aberrantly accumulated endogenous substances. There are some reviews that provide an in-depth introduction to macrocyclic host-based sequestration agents.^21,72^

**Figure 2:**
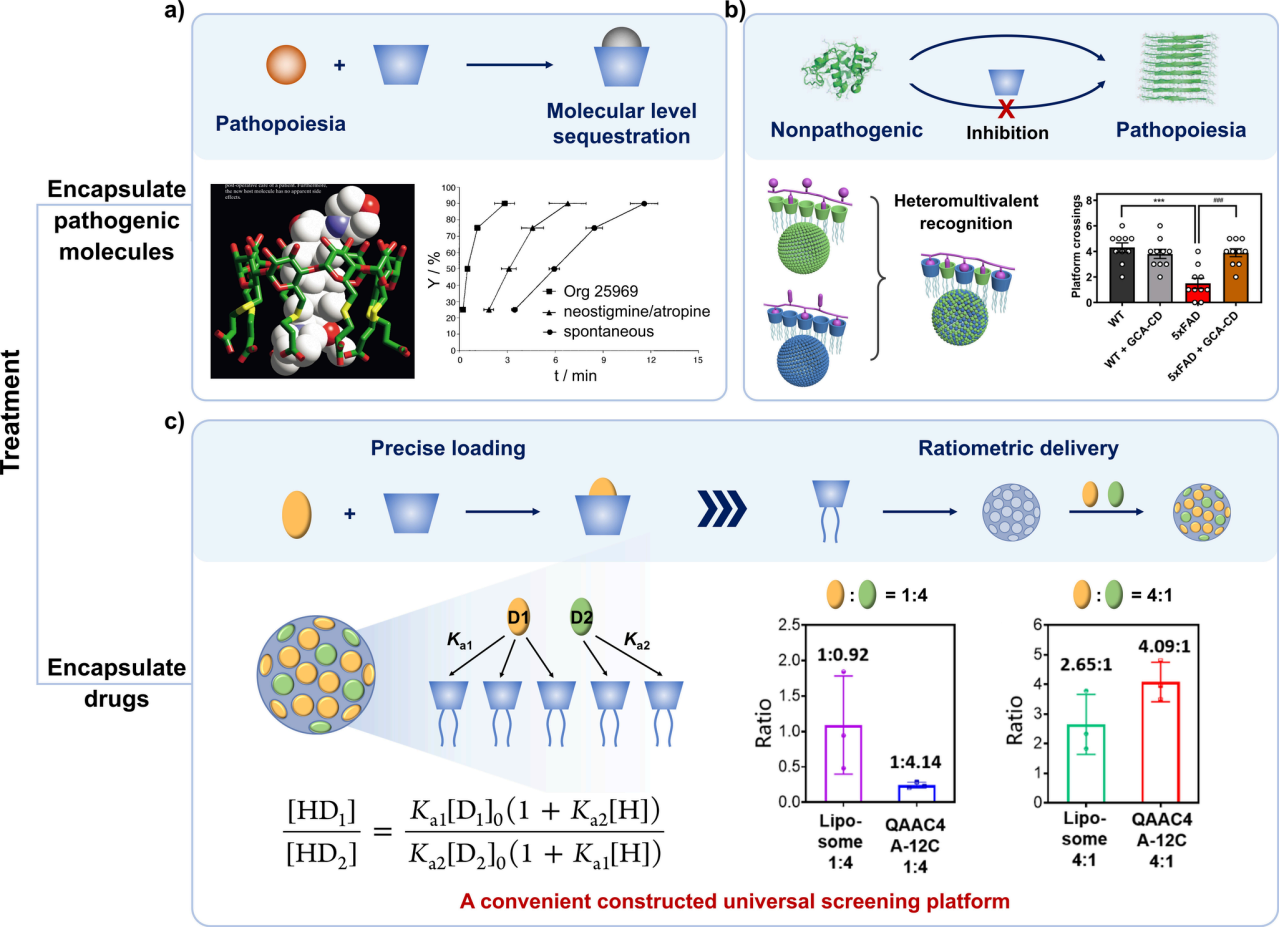
Applications of macrocyclic hosts in disease treatment through encapsulating pathogenic molecules or drugs. (a) Schematic diagram illustrating the alleviation of toxicity of pathogenic molecules by macrocyclic hosts (upper), and an example of using Sugammadex to reverse the nerve block caused by rocuronium (bottom). Adapted with permission from ref 73. Copyright 2002 Wiley-VCH GmbH. (b) Schematic diagram illustrating the alleviation of toxicity of misfolded peptides/proteins by macrocyclic hosts (upper), and an example of using the coassembly of CD and CA to inhibit the Aβ fibrillation thereby improving cognitive impairments in Alzheimer’s disease mice (bottom). Adapted with permission from refs 76 and 77 . Copyright 2019 Springer Nature. Copyright 2020 Chinese Chemical Society. Distributed under a Creative Commons Attribution-NonCommercial 3.0 Unported license. (c) Schematic diagram of precise loading and ratiometric delivery of drugs by macrocyclic hosts and an example of using CA-based nanocarrier to deliver two drugs in molar ratios of 1:4 and 4:1. Adapted with permission from ref 85. Copyright 2021 Wiley-VCH GmbH.

There are approximately 50 diseases associated with the misfolding of peptides and proteins. Inhibiting and reversing the misfolding of peptides/proteins is a promising approach for treating these diseases, and the key to this approach is the development of inhibitors that selectively and tightly bind to peptides/proteins.^74^ Lots of macrocyclic hosts and their derivatives can complex with amino acid residues, therefore have made significant contributions in this field.^75^ For the amyloid-β (Aβ) peptide, whose fibrillation is the hallmark of Alzheimer’s disease, our group has developed a coassembled heteromultivalent recognition strategy to handle its large size and diverse binding sites.^76^ By coassembling different amphiphilic macrocyclic hosts, we obtained the nanoparticle that has diverse recognition sites on its surface and thus can simultaneously bind multiple and different amino acid residues on Aβ (Figure 2b). The coassembly (CD-GCA) composed of amphiphilic cyclodextrin (CD12C, Scheme 1) and guanidinium-modified calixarene (GC5A12C, Scheme 1) achieved ultrastrong binding to Aβ_42_ with the binding constant reaching up to 10^13^ M^–1^.^77^ It is worth noting that the binding strength even surpasses that of currently marketed antibodies.^78^ An objective fact is that these antibodies are more focused on binding oligomers and fibrils but not Aβ monomers. From an enthalpy perspective, the macrocyclic host provides a hydrophobic microenvironment, leading to stronger interactions with the binding sites. From an entropy perspective, the complexation of coassemblies with Aβ peptides may give rise to less conformational freedom loss. Benefiting from the strong affinity, CD-GCA almost completely inhibited Aβ fibrillation and rapidly disintegrated the preformed fibrils, effectively improving cognitive impairments in Alzheimer’s disease mice (Figure 2b). The coassembled heteromultivalent recognition is a platform strategy that simply necessitates selecting the optimal types and ratios of coassembling hosts tailored to different peptides.^79^ We have also extended to use coassemblies to recognize proteins. We achieved strong binding toward the α-synuclein using coassembly, inhibiting its fibrillation and thereby enabling effective intervention in Parkinson’s disease.^80^ However, we noticed that the coassemblies were more suitable to recognize peptides rather than proteins. For example, the binding strength of CD-GCA toward bovine serum protein, trypsin, β-galactosidase, and ubiquitin, were over 6 orders of magnitude lower than that toward Aβ_42_.^77^ This selectivity arises from the mismatch between the protein’s inherent three-dimensional structure and the spherical configuration of the assembly, rendering many residual sites inaccessible to the coassemblies. One more reasonable approach for protein recognition was envisaged to build heteromultivalent recognition receptors based on flexible polymer scaffolds. In addition to (synergistically) complexing with amino acid residues, some giant macrocyclic hosts, such as biphenarenes, can also envelop peptides as whole, showing tremendous potential in inhibiting peptide fibrillation.^81^

Macrocyclic hosts can not only treat diseases by recognizing pathogenic molecules, but also deliver drugs to the site of lesions by recognizing and loading drugs, thereby treating diseases.^82,83^ Macrocyclic host-based drug delivery systems (DDSs) exhibited lots of advantages, among which the most notable is their precise drug loading capability (Figure 2c).^84^ Macrocyclic carriers have well-defined structures, exact cavity-loading pattern, and quantifiable host-drug binding constant.^24^ The host–guest complexation kinetics is quite rapid (the dissociation rates are commonly on the microsecond to millisecond time scale),^53^ which can effectively avoid the generation of kinetic traps, ensuring that the drug loading process is thermodynamically controlled. That means that when equilibrium is reached, the concentration ratio of the formed complex (loaded drug) to the concentration of the free host and unloaded drug will always be a constant (binding constant). Based on this, our group has derived equations for calculating the drug loading efficiency (LE) and entrapment percentage (EP).^84^ Using initial mixing concentrations of the macrocyclic carrier and the drug, as well as the binding constant between them, we can calculate the LE and EP. Similarly, by altering these parameters, we can adjust the LE and EP. This enables us to administer drugs as needed, meeting the requirements of precision medicine and personalized medicine. Furthermore, this precise loading model can also be extended to ratiometric drug loading and delivery. In combination therapy, different drug ratios may result in varying therapeutic effects, synergistic or antagonistic. Therefore, the development of a convenient, universal, and precise drug ratio screening tool is highly on demanded. Amphiphilic macrocyclic carriers can spontaneously assemble into aggregates and provide surfaces with multiple binding sites, endowing the simultaneous precise loading of two or more drugs. The loading ratios can be predicted, adjusted and reproduced, and drugs can be ratiometric delivered to the lesion site (Figure 2c).^85^ Moreover, the macrocyclic hosts can encapsulate a broad spectrum of drugs, making them adaptable to different diseases and treatment modalities.^86^ Therefore, macrocyclic hosts provide an efficient and universal tool for optimal ratio screening in combination therapy. In addition, macrocyclic host-based DDSs also have many other advantages.^87^ First, they are easy to construct because the host–guest recognition is spontaneous; the loading can be achieved by simply mixing the drug with the host. Second, macrocyclic hosts can offer molecular-level steric barriers that, in favorable cases, improve the physicochemical properties of drugs, such as increasing solubility and stability and reducing side effects. Third, macrocyclic host-based DDSs do not require the modification of drugs, making them suitable for various drugs and enabling traceless release of drugs. Moreover, due to the ease of construction and universality toward drugs, macrocyclic host-based DDSs can be tailored for patient-specific treatment.

### 3.2 Exerting Functions through Frameworks

Macrocyclic hosts not only demonstrated biomedical functions by recognizing bioactive molecules but also inherently possess biological activities that contribute to disease diagnosis and treatment. However, their diagnostic applications are relatively limited, primarily involving the attachment of imaging groups to the macrocyclic skeletons.^88^ In contrast, their therapeutic potential is more pronounced. Consequently, this section will concentrate on the therapeutic applications of macrocyclic frameworks, categorized into two cases: 1. the interaction between macrocyclic frameworks with proteins and membranes, 2. the inherent reducibility of electron-rich macrocyclic frameworks. In addition to these two cases, macrocyclic hosts also provide a scaffold for drugs/functions integration.^89^ However, this is not a domain where macrocyclic hosts particularly distinguish themselves, as other drug/function integrated platforms, like polymers and dendrimers, have already excelled in this regard. Therefore, we will not elaborate extensively on the applications of macrocyclic hosts in this context.

An effective method for disabling anthrax lethal toxin is to design a cationic low-molecular-weight nonpeptide compound to block the pore of PA_63_. The pore of PA_63_ has a diameter of ∼20 Å and exhibits a 7-fold symmetry. This easily brought researcher’s mind to β-CD, which has a diameter of ∼15 Å and the same symmetry (Figure 3a).^90^ According to the structure of PA_63_ pore, 7 positive charges were further introduced on β-CD. These β-CD derivatives formed strong complexes with the PA_63_ pore, with the binding constant as high as 10^9^ M^–1^, thereby impeding the normal function of PA_63_ and effectively alleviating the toxicity of anthrax lethal toxin. The most effective β-CD derivative, AMBnTβ-CD (Scheme 1), completely protected Fisher F344 rats from intoxication with lethal toxin and, in combination with the antibiotic ciprofloxacin, significantly increased the survival of mice in an infection model of anthrax.^22^ In addition, a series of C4A derivatives (Scheme 1) have been reported as novel HIV-1 integrase inhibitors, because they can be encapsulated into the hydrophobic cavity formed by HIV-1 integrase amino acid residues.^91^ Another C4A derivative, 0118 (Scheme 1), has been reported to interact with galectin and attenuates its binding to cell surface glycans, thereby inhibiting the galectin-1-mediated cell agglutination and cell proliferation.^92^ Compared to other small molecule drugs that exert therapeutic effects by interacting with proteins, macrocyclic hosts have larger sizes and special three-dimensional structure. This makes them potentially better matched with certain sites, leading to a higher efficiency in impacting functions of proteins.

**Figure 3:**
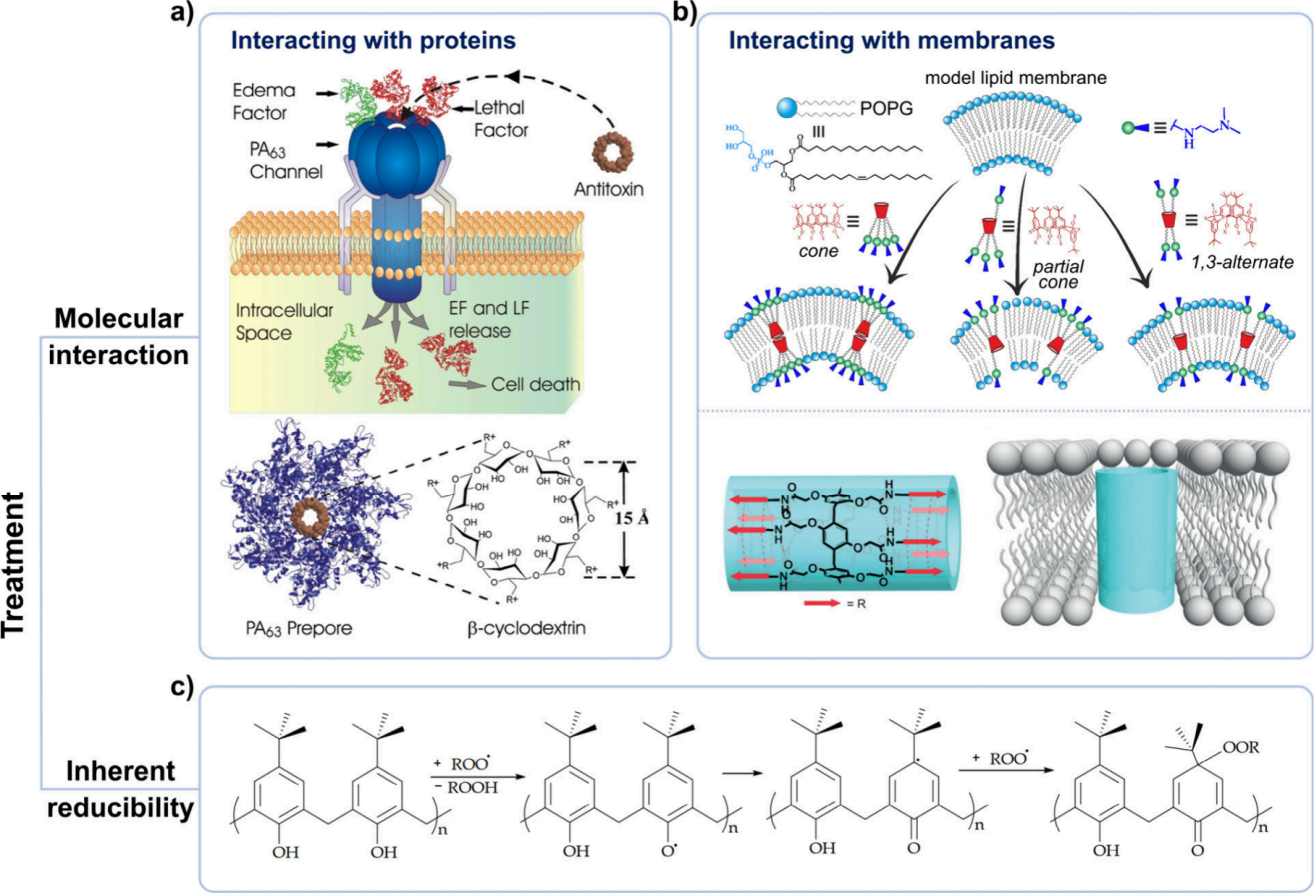
Applications of macrocyclic hosts in disease treatment through interact with proteins/membranes or inherent reducibility. (a) Schematic diagram of using β-CD to block the pore of PA_63_, thereby disabling anthrax lethal toxin. Adapted with permission from ref 90. Copyright 2010 Biophysical Society. (b) Upper: Schematic diagram illustrating the effect of the CA conformation on its interaction with the membrane. Adapted with permission from ref 98. Copyright 2023 DMPI. Distributed under the terms and conditions of the Creative Commons Attribution license. Bottom: Schematic diagram showing the formation of channels by PA on the membrane. Adapted with permission from ref 103. Copyright 2017 Wiley-VCH GmbH. (c) Schematic diagram of the scavenging of reactive oxygen species by the CA.

In addition to interacting with proteins, macrocyclic host derivatives can also interact with biological membranes, affecting fluidity and permeability of the membrane, thereby exhibiting therapeutic activities such as antimicrobial and antitumor.^93–95^ Among macrocyclic hosts, CAs and PAs are more commonly used in this field because they are easier to be modified. For CAs, their upper and lower rims can be easily modified with different groups, facilitating the development of amphiphilic molecules. Currently, over 400 amphiphilic CAs have been reported.^96^ By alteration of the types of hydrophilic (hydrophobic) groups on the upper rim and hydrophobic (hydrophilic) groups on the lower rim, the membrane-disrupting activity of amphiphilic CAs can be finely adjusted. Many well-designed amphiphilic CAs have exhibited antibacterial activity while maintaining high safety for normal cells.^97^ Moreover, the framework of CA plays a crucial role in enhancing its antimicrobial activity. First, under the same modifying groups, the CA in a partial cone structure can show better antibacterial activity compared to CAs in cone and 1,3-alternate structures. This may be attributed to the asymmetric nature of the partial cone CA, which enhanced its membrane-disrupting capability (Figure 3b).^98^ Second, even for CAs in cone structure, their antimicrobial activities can be much higher than those of monomers. For example, when against fungal strains, the iminecalix[4]­arene (IC4A, Scheme 1) showed the minimal inhibitory concentrations ∼ 36 times lower than its monomers, exceeding the increase expected from the number of repeating units.^99^ For PAs, asymmetric modifications are not as easy as CAs, however, their unique pillar-like structure makes it easy to construct tubular molecules that can insert into the lipid bilayer to form transmembrane channels.^45,100–102^ Prof. Hou reported that tryptophan-incorporated peptide modified PAs displayed high insertion selectivity for the lipid bilayer membrane of Gram-positive bacteria over that of the mammalian erythrocytes, thereby disrupting the homeostasis of the internal environment of Gram-positive bacteria, showing high antimicrobial activity and low hemolytic toxicity (Figure 3b).^103^

As electron-rich macrocyclic hosts with multiple phenolic hydroxyl groups, CAs (including resorcinarenes) exhibit strong reducing properties and are able to scavenge various reactive oxygen species (ROS) and safeguard normal tissues (Figure 3c).^104,105^ Therefore, they have achieved successes in the treatment of a series of inflammatory-related conditions, such as rheumatoid arthritis, bacterial infection, and Alzheimer’s disease.^106–109^ Again, the macrocyclic framework plays a significant role in the antioxidant capacity; the efficiency of CA in ROS scavenging can be higher than that of their constituent repeating units (computed based on the equivalent of repeating units). For example, tetra-(3,5-dihydroxy)­styryl-C4A (TSC4A, Scheme 1), a tetramer of resveratrol, was reported to have remarkably lower 4′–OH bond dissociation enthalpies, ionization potential, and 4′-phenolate proton affinity than those of resveratrol, indicating that TSC4A exhibits much more excellent antioxidative activity than resveratrol.^110^ Spin density distribution analysis proved that the 4′-phenoxyl radical and cation radical of TSC4A possessed a higher degree of unpaired electron delocalization with respect to the corresponding radical species of resveratrol. Topologies of orbitals occupied by unpaired electron shows that the CA framework plays a very important role in delocalization of unpaired electron, thus making the phenoxyl radical and cation radical of TSC4A much more stable than that of resveratrol. Additionally, the antioxidant capacity of CAs can also vary depending on the number of repeating units. For example, Prof. Gorghiu reported that the antioxidant feature of *p*-*t*-butyl-C4A is higher than that of *p*-*t*-butyl-C6A and *p*-*t*-butyl-C8A.^104^ Moreover, the ease of modification of CAs provides opportunities for continuously optimizing their antioxidant capabilities.

### 3.3 Exerting Functions through both Recognition and Framework

#### 3.3.1 Diagnosis

Amphiphilic macrocyclic hosts have attracted significant attention in the construction of Förster Resonance Energy Transfer (FRET) imaging systems.^111,112^ Deemed as “surfactants with host-guest recognition sites”, macrocyclic hosts can self-assemble into supramolecular aggregates, providing discrete loading sites for donors and acceptors, i.e. outer cavity encapsulation and inner hydrophobic entrapment.^111^ Therefore, FRET systems based on macrocyclic hosts do not require chemical modification of the luminophores (donor/acceptor), can accommodate various luminophores, and allow for convenient adjustment of their ratios. These systems have been utilized for imaging cancer cells, showcasing advantages such as low background noise and spatial consistency of signals with the target analytes, especially when compared to the common imaging method, IDA.^113^ Additionally, FRET systems are adaptive for imaging the autophagy process from the perspective of space. The key step in autophagy involves a continuous approach and fusion between autophagosomes and lysosomes. While in FRET systems, the energy transfer efficiency between donor and acceptor is highly sensitive to their spatial distance. Our group has recently reported a FRET autophagy imaging platform by leveraging both recognition and assembly properties of macrocyclic amphiphiles (Figure 4a).^114^ The amphiphilic sulfonated C6A (SC6A12C, Scheme 1) was coassembled with lysosome-targeting *N*-cetylmorpholine to create a supramolecular FRET carrier (SN). Then the FRET donor (9,10-Bis­(2-phenylethynyl) anthracene) was loaded in the hydrophobic region of SN. The cavity of SC6A12C can bind various FRET acceptors, including Mito-Tracker Red, ER Tracker Red, and RhoNox-1, which target mitochondria, endoplasmic reticulum, and Golgi apparatus, respectively. The cells were sequentially incubated with donor-SN and acceptor, and they accumulated within their respective targeted organelles. If there was autophagy, then the degradation of organelle membranes provided the opportunity for spatial proximity of FRET donors and acceptors, driven by the host–guest complexation.

**Figure 4:**
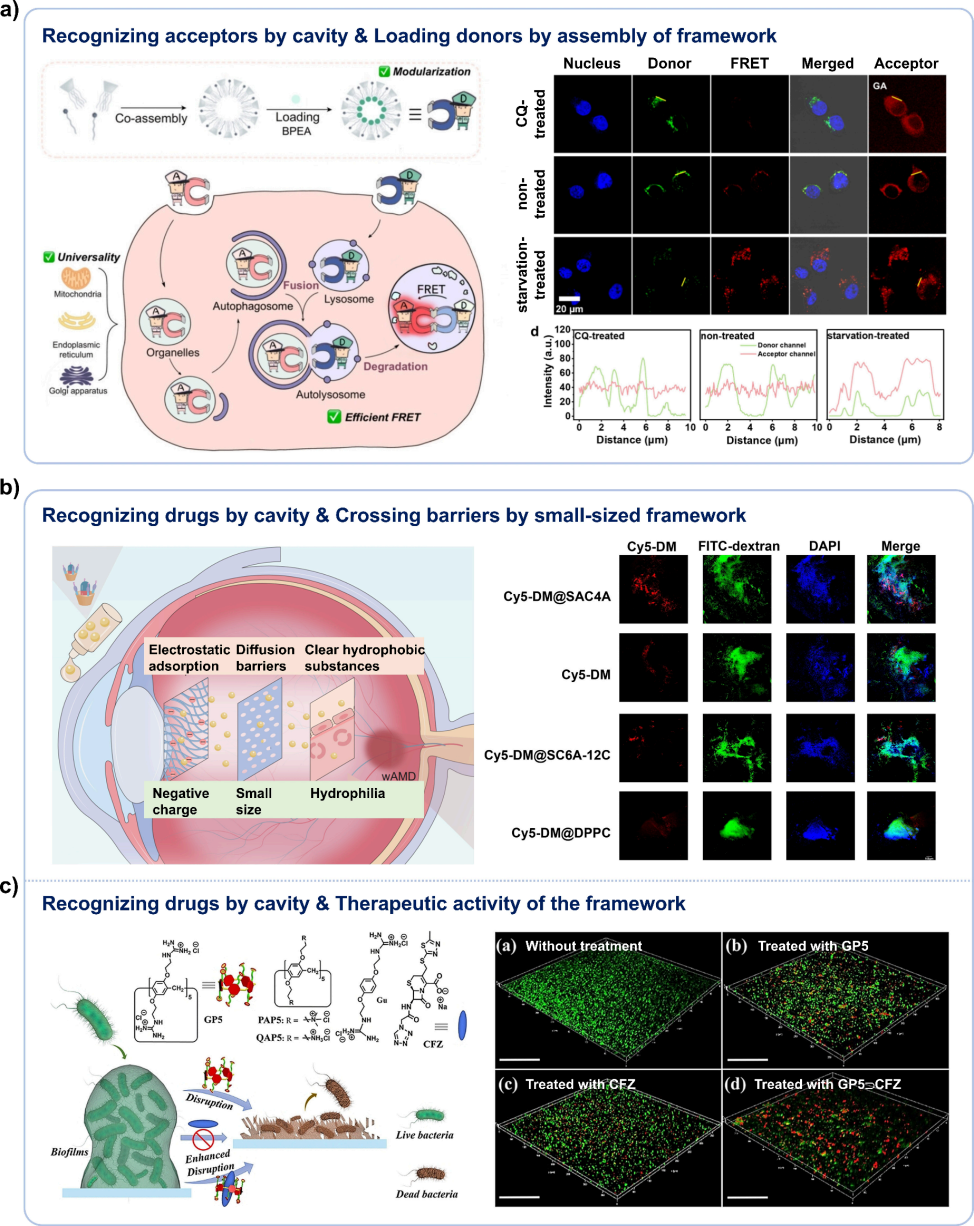
Biomedical applications of macrocyclic hosts through recognition and framework simultaneously. (a) Schematic diagram of the construction of a FRET imaging system for autophagy processes by recognizing acceptors by cavity and loading donors by assembly of the amphiphilic CA (left), as well as the imaging results (right). Adapted with permission from ref 114. Copyright 2025 Wiley-VCH GmbH. (b) Left: Illustrative diagram showcasing drug delivery to the posterior segment of the eye by recognizing drugs by the cavity of SAC4A and utilizing the barrier-crossing ability of its small-sized framework. Right: The delivery efficient of model drugs to the posterior segment of the eye. Adapted with permission from ref 119. Copyright 2024 Elsevier. (c) Schematic diagram illustrating the synergistic therapeutic effect of the drug loaded in the GP5 cavity and the framework of GP5 in disrupting the biofilm (left), as well as the treatment effects (right). Adapted with permission from ref 120. Copyright 2021 Wiley-VCH GmbH.

#### 3.3.2 Treatment

In drug delivery, macrocyclic hosts often load drugs within their cavities while simultaneously leveraging the biological activity of their framework, such as crossing barriers and exerting therapeutic activity, to enhance the effectiveness of disease treatment. Over the years, it has been repeatedly discovered that even negatively charged CAs, such as sulfonated calix[4]­arene, carboxyl-modified azocalix[4]­arene, could effectively enter cells and transport positively charged small molecules and peptides that would otherwise hardly to enter.^115–118^ Moreover, their effective concentrations to achieve half maximal activity can be ∼1000 times lower than that of pyrenebutyrate (gold standard) and ∼100 times lower than that of corresponding monomer, indicating their remarkably high efficiency.^117^ We speculate that the excellent transmembrane transport capability of CAs may be due to their cycle structure, which can lead to significant membrane perturbation upon interaction. In addition to biomembranes, macrocyclic carriers also demonstrate remarkable proficiency in penetrating other barriers. The size of macrocyclic carrier can be extremely small compared with other nanocarriers, in the range of hundred Å^3^, which is advantageous for traversing majority of biological barriers. Moreover, macrocyclic carriers can be tailored through rational modifications to adjust their hydrophilicity and electronic properties according to requirements, thereby achieving enhanced penetration through barriers. For example, sulfonated azocalix[4]­arene (SAC4A, Scheme 1) has been applied for penetrating ocular barriers and delivering drugs to the ocular posterior segment (Figure 4b).^119^ Its small size imparts a high diffusion rate to successful passage of the dense structure of the eye. The negative charge and appropriate hydrophilicity enable it to evade adsorption by negatively charged tear film, sclera, and vitreous, while also circumventing rapid clearance by the retinal.

The macrocyclic hosts can also leverage their intrinsic therapeutic activities while delivering drugs, thereby enabling combination therapy for diseases. For example, Prof. Wang reported using a complex of cefazolin sodium (CFZ) with guanidinium-functionalized pillar[5]­arene (GP5, Scheme 1) to treat bacterial infection.^120^ GP5 exhibited high activity in disrupting both preformed biofilms and bacterial membranes. When working together with the loaded antibiotic CFZ, the complex can efficiently penetrate through biofilms, breach the bacterial membranes, reach the periplasm, and kill the bacteria synergistically (Figure 4c). Our group has prepared a complex of hydroxychloroquine (HCQ) with SAC4A to treat rheumatoid arthritis.^106^ SAC4A acted as a hypoxia-responsive carrier, delivering and releasing HCQ into the inflammatory articular cavity. At the same time, it also exhibited a ROS scavenging capacity and served as an anti-inflammatory agent. Compared with individual HCQ or SAC4A treatments, the complex HCQ@SAC4A showed better therapeutic effects.

## 4 Future Opportunities and Challenges

The macrocyclic hosts have undoubtedly provided efficient and unique building blocks for diagnostic and therapeutic materials. Their extensive chemical design space and modular construction characteristics enable them to be suitable for a variety of scenarios. Moreover, macrocyclic hosts have exhibited some unparalleled properties. For example, benefiting from their preorganized cavities, macrocyclic hosts can recognize bioactive substances with high affinity, enabling effective intervention in biological processes and precise drug loading.^121^ Benefiting from their unique framework, macrocyclic hosts show high efficiency in crossing barriers, interacting with proteins/membranes, etc.^22^ Herein, we express some of our insights on the future opportunities of macrocyclic hosts and discuss the challenges it faces in leveraging these advantages along with suggestions for overcoming these challenges (Figure 5).

**Figure 5:**
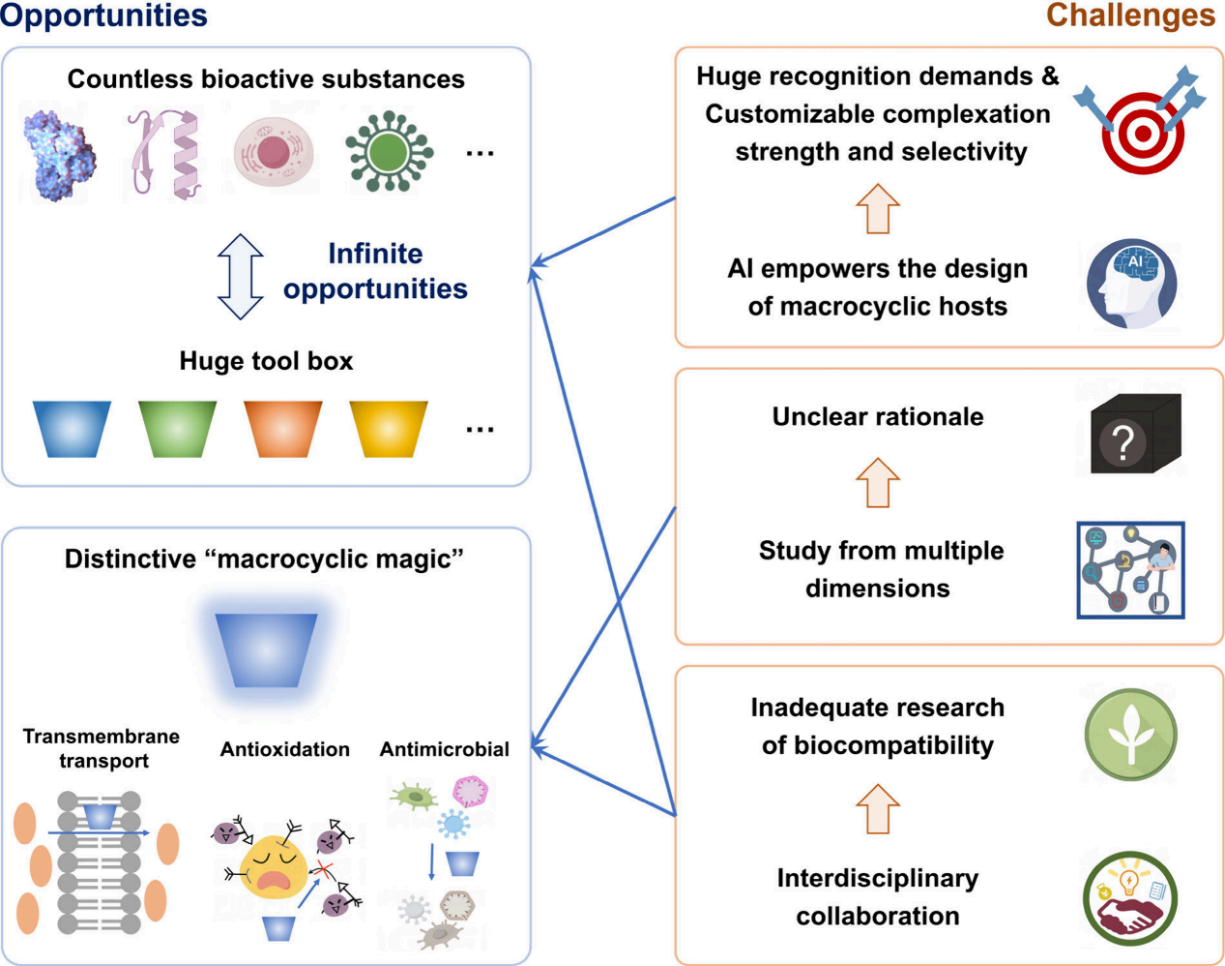
Some of our insights on the future opportunities and challenges of macrocyclic hosts in the field of biomedical applications.

First, serving as alternatives to natural recognition systems as recognition tools, macrocyclic hosts hold great promise in the field of biomedicine. Recognition is a common and crucial process for organisms to maintain normal life activities; therefore, macrocyclic host-based biomedical materials have great potential in understanding, mimicking, and intervening biological processes. There are countless bioactive substances in nature, including small molecules, biomacromolecules, cells, microorganisms, etc., providing a huge recognition demand as well as opportunities. At the same time, a rich library of macrocyclic hosts, including blue boxes, heteracalixaromatics, coronarenes, helicarenes, octopusarenes, naphthotubes, biphenarenes, corralarenes, etc. has been established and continues to expand.^38^ These macrocyclic hosts each have their own characteristics, such as intrinsic chirality and an extremely large cavity, offering a powerful toolkit for the recognition of bioactive substances. Compared to natural receptors such as antibodies and enzymes, macrocyclic hosts have also achieved strong binding to certain bioactive substances, but selectivity is not comparable yet. However, it is precisely such “semi-selective” recognition of macrocyclic hosts that advances in areas such as the delivery of broad-spectrum drugs and simultaneous detection of multiple biomarkers. However, current research on macrocyclic host recognition is still limited. According to the Suprabank database, supplemented by recent literature, there are only over 6000 reported data points, with even fewer examples of strong binding. Instances of binding affinities exceeding 10^6^ M^–1^ account for only 15.1% of the total data set.^121^ Moreover, these recognitions are not exclusively toward bioactive substances. This greatly impacts the development of macrocyclic host-based biomaterials. The inadequate recognition strength can lead to high detection limits in diagnostics, poor therapeutic effects in treatment, and off-target leakage in drug delivery. Moreover, inadequate recognition selectivity makes it difficult for macrocyclic hosts to achieve their biological functions in the presence of endogenous interferents. However, it is unable to meet the huge recognition demands by relying on a labor-intensive approach of trial and error to screen/design macrocyclic hosts for bioactive substances individually. There is a need to change the paradigms. The prevalence of artificial intelligence (AI) has brought a new perspective to the design of macrocyclic hosts. Collaboratively employing experimental titration, computational simulation, and machine learning, we are hopeful to establish a prediction model for host–guest recognition and further guide the screening and design of macrocyclic hosts. The premise for achieving these goals lies in the establishment of a comprehensive and standardized database, thereby propelling the field of AI-driven supramolecular chemistry and materials forward.

The semi-selective recognition of macrocyclic hosts with bioactive substances provides significant advantages in various bio-applications, especially in areas such as the delivery of broad-spectrum drugs and simultaneous detection of multiple biomarkers.

Second, the framework of the macrocyclic host displays a distinctive “macrocyclic magic”, showcasing high effectiveness in several applications, such as transmembrane transport, antioxidation, and antimicrobial, which are notably superior to monomers, surpassing the effect achieved solely by increasing concentration. As we previously mentioned, amphiphilic sulfonate C4A exhibited a transmembrane transport efficiency ∼100 times greater than its monomer.^117^ TSC4A exhibited much more excellent antioxidative activity than resveratrol.^110^ IC4A exhibited minimal inhibitory concentrations approximately 36 times lower than its monomers.^99^ These examples all illustrate that macrocyclization greatly enhances the bioactivity of the monomers, while this may be due to not only increased local concentration but also a multivalent effect. In addition, the “macrocyclic magic” can also lead to better safety. Amphiphilic macrocyclic hosts have much lower aggregation micelle concentration and denser assembly than their corresponding monomers.^96^ Therefore, they have a greater tendency to self-assemble without disrupting biological membranes, thus exhibiting a better biocompatibility. The unique magic of macrocyclic frameworks endows the macrocyclic host with significant potential for developing efficient and safe biomedical materials. However, the rationale behind the “macrocycle magic” has not been thoroughly understood. Although significant efforts have been made, research in this area remains fragmented. This may result in the potential of macrocyclic frameworks not being fully utilized. In fact, we first need to focus on in which applications macrocyclic hosts exhibit the “macrocyclic magic”, besides the applications mentioned earlier, whether there are other applications where this magic is also observed. We believe that in previous research this aspect has not received enough attention. In addition, we need to study the “macrocyclic magic” from multiple dimensions, ranging from the physicochemical properties of the hosts to their structure–activity relationships to mechanisms of their bioactivities, delving deeper at each level. This necessitates collaborative efforts from scientists across various disciplines, such as chemistry, materials science, physics, and life sciences.

The frameworks of macrocyclic hosts exhibit magic biomedical functionalities, extraordinarily surpassing the activities of their corresponding monomers, which potentially offer fresh perspectives and avenues for designing biomedical materials.

Additionally, there is another challenge affecting the biomedical applications of macrocyclic hosts; that is, the research on their biocompatibility is not yet systematic and thorough enough. Although there is a plethora of various biomedical applications based on different macrocyclic hosts in basic research, the number of those that have entered the clinical trial is less than 100, with the vast majority being based on CDs (www.clinicaltrials.gov). The insufficient research on biocompatibility is one of the key reasons for this disappointing bench-to-bedside translation rate. In addition to the typical tests like blood analysis, blood chemistry, and histopathologic analysis of major organs, other aspects including biodistribution, metabolism, genotoxicity, maximum tolerated dose, acute toxicity, long-term toxicity, immunogenicity, reproductive toxicity, toxicity of the metabolites, etc. also need to be investigated. However, conducting systematic research on the biocompatibility of macrocyclic hosts is cost- and time-intensive, which is almost beyond the capacity of most research groups. Therefore, extensive collaboration with experts in pharmacology, medicine, and companies is necessary.

The deep integration of experimentation, computational simulation, and AI would better serve the application of macrocycles in biomedicine.

## 5 Conclusion

Macrocyclic hosts, with preorganized recognition sites and unique frameworks, have been widely applied in the diagnosis and treatment of diseases, demonstrating numerous advantages. While significant progress has been made, we believe that macrocyclic hosts still hold vast untapped potential for further exploration and development. This Outlook provides a glimpse into the rich biomedical applications and unique properties of macrocyclic hosts. We hope that it not only offers new ideas and directions for supramolecular chemists but also draws the attention of scientists from other fields to the potential of macrocyclic hosts. Through collaborative efforts, we aim to enhance the role of macrocyclic hosts in advancing biomedical research, facilitating the transition of more macrocyclic hosts from cutting-edge research to clinical applications.
